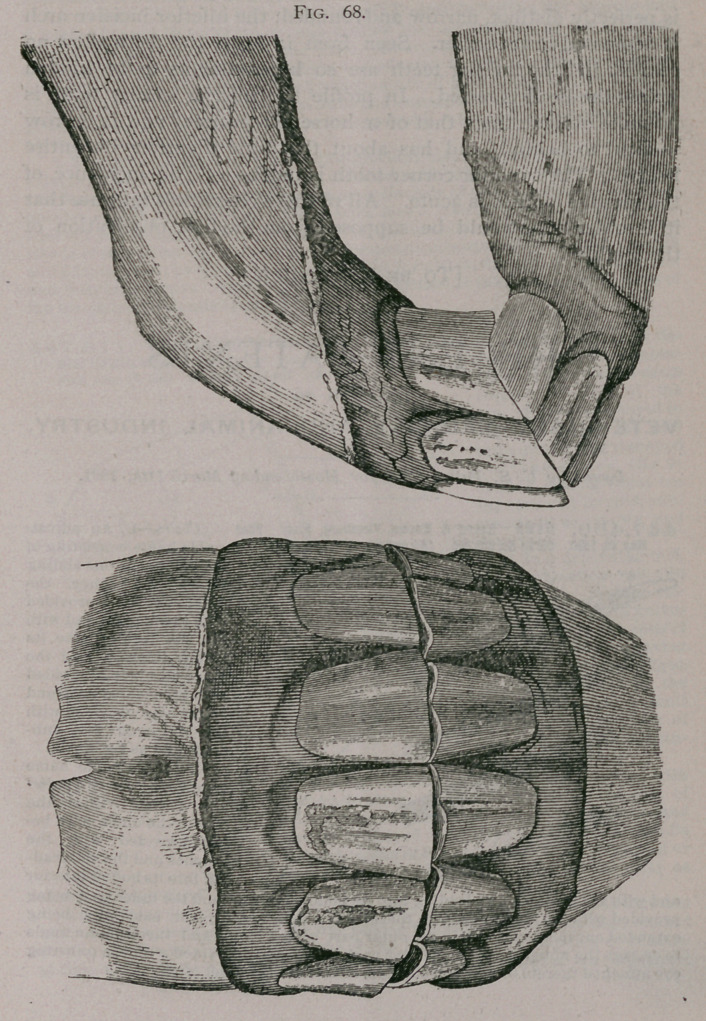# Age of the Horse, Ox, Dog, and Other Domesticated Animals

**Published:** 1891-04

**Authors:** R. S. Huidekoper

**Affiliations:** Veterinarian


### AGE OF THE HORSE, OX, DOG, AND OTHER DOMESTICATED ANIMALS.

By R. S. Huidekoper, M.D., Veterinarian.

[*Continued from page 121.*]

Fig. 61.

Figures 61, 62, show an upper jaw from the collection of Dr. J. W. Gadsden, of Philadelphia, in which will be seen—supernumerary incisor; supernumerary intermediate teeth; corner teeth of first dentition still remaining.

Fig. 62.

Fig. 63.

Figure 63 represents a sub-zygomatic molar, natural size, removed from a three months old gelding, by Dr. W. J. Martin, of Kankakee, Kansas.

### Irregularities of Form.

In certain subjects the incisors of the lower jaw present, at the age of six years, a decided triangular form such as is usually seen at 14 to 15 years of age.

This triangularity is readily distinguished from that of the older age by the presence of the cup.

### Irregularities by Uniting of two Incisors.

This sort of irregularity has no special importance as to the determination of age, but is interesting as a curiosity. Figure 64 shows the upper jaw of a horse with a double left hand intermediate tooth, in which the two cups are perfectly distinct.

FIG. 64.

IRREGULARITIES IN FORM OF THE DENTAL CUP.—FISSURE.

Figure 65, 66. Fissure of the incisor teeth, by failure of the enamel on the posterior face, to completely surround them, is

FIG. 65.

FIG. 66.

rather more common in asses and mules than in horses. The fissure may represent only a small portion of the tooth, or it may constitute a complete division of the posterior surface, leaving the peripheral enamel in the form of a crescent.

### IRREGULARITIES IN DEPTH OF THE DENTAL CUPS.

Frequently the dental cup continues on the table of the teeth at a time when it should have disappeared. This occurs more frequently in very well-bred horses in which the development of the teeth has been precocious and in whom the teeth have such a consistency and hardness that they do not wear away by use, as in more common-bred horses.

### EXCESS OF HARDNESS OF THE TEETH.

The nature of the food it has eaten and the condition of health of the teeth are all causes which tend to influence this irregularity. It is a condition rarely noticed before the age of seven or eight years..

The length of the teeth and the form of the dental tables, is less to be considered in estimating successive length of the teeth, than is the form of the dental tables and that of the cups.

### EXCESS OF LENGTH OF THE DENTAL CUP.

Sometimes while the teeth have worn in the ordinary way, have diminished in length, and have undergone the usual changes in the shape of their tables, the cups continue in size and shape, not corresponding with the other indications of age. In these an attentive examination of the tables of the incisors with their shape, the size of the dental star, its situation, the condition of the upper incisors, the direction of the teeth, their length, color, etc. will rectify the faults of the cups.

FIG. 67.

FIG. 67.

Figure 67. This plate represents the teeth of a horse nine years of age; the dental cups are of a size and shape representing a younger age. In Profile the mouth is that of a horse five or six years of age; the inferior tables are round, the central enamel are close to the posterior border of the teeth. The corner teeth are

FIG. 68.

levelled and rounded, the dental star is near the middle of the teeth. These characters added to the freshness of the corner teeth, the obliquity of the inferior incisors, the notch on the upper corners, and the general condition of the subject, are sufficient to rectify any error indicated by the first inspection.

FIG. 68.

Figure 68. This figure is taken from the mouth of a horse fourteen years of age. The levelling of the teeth would only indicate about ten years of age but a close examination of the tables show: 1st, that the pincers are nearly triangular; 2nd, the intermediate teeth commence to become so; 3rd, the dental star is perfectly distinct, narrow and rounded; the inferior incisive arch is depressed in its center. Seen from in front, the teeth show no cement and the inferior teeth are so horizontal as to be hidden unless the head is raised. In profile the inferior corner tooth is no more oblique than that of a horse ten years of age, is narrow in front to behind, and has about the same diameter its entire length. The superior corner tooth is notched. The incidence of the incisive arches is acute. All of these characters indicate that it is older than would be supposed from the first observation of the teeth.

[To be Continued.]

## Figures and Tables

**Fig. 61. f1:**
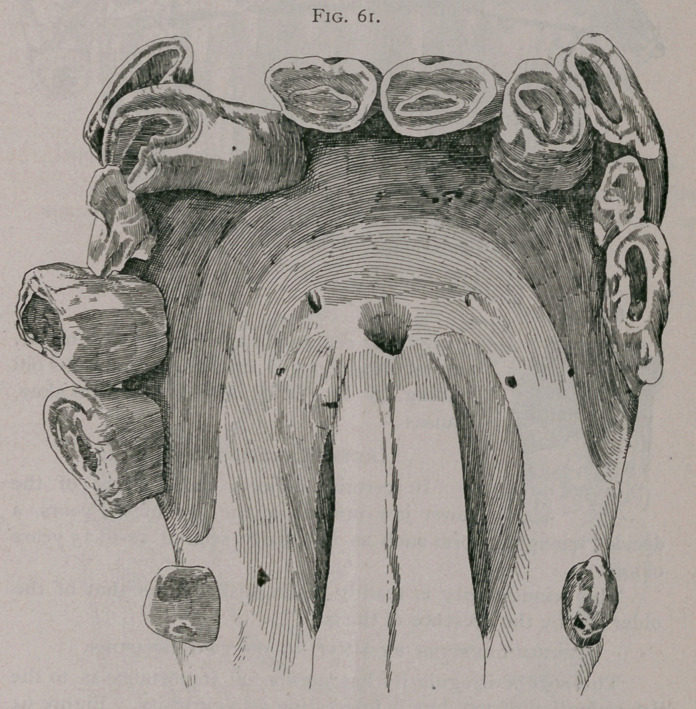


**Fig. 62. f2:**
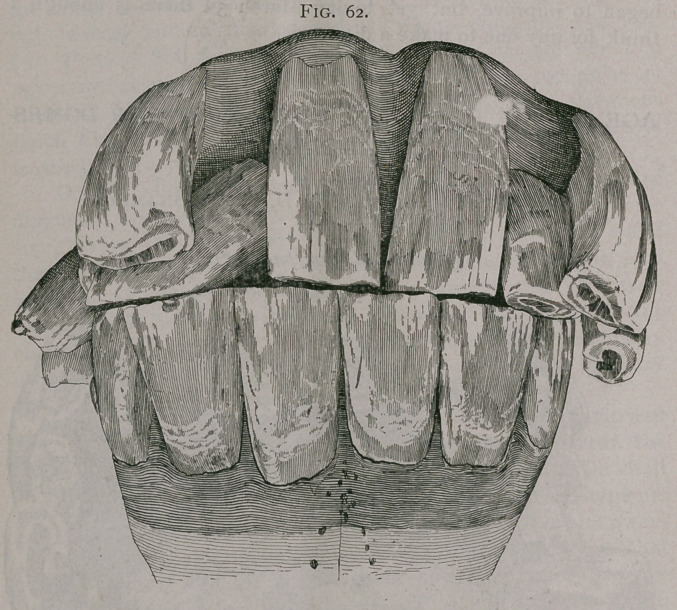


**Fig. 63. f3:**
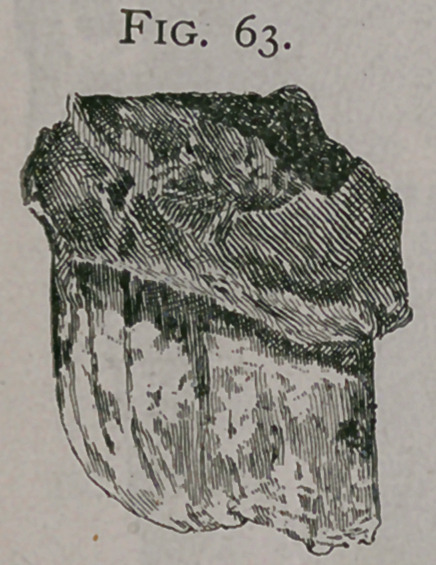


**Fig. 64. f4:**
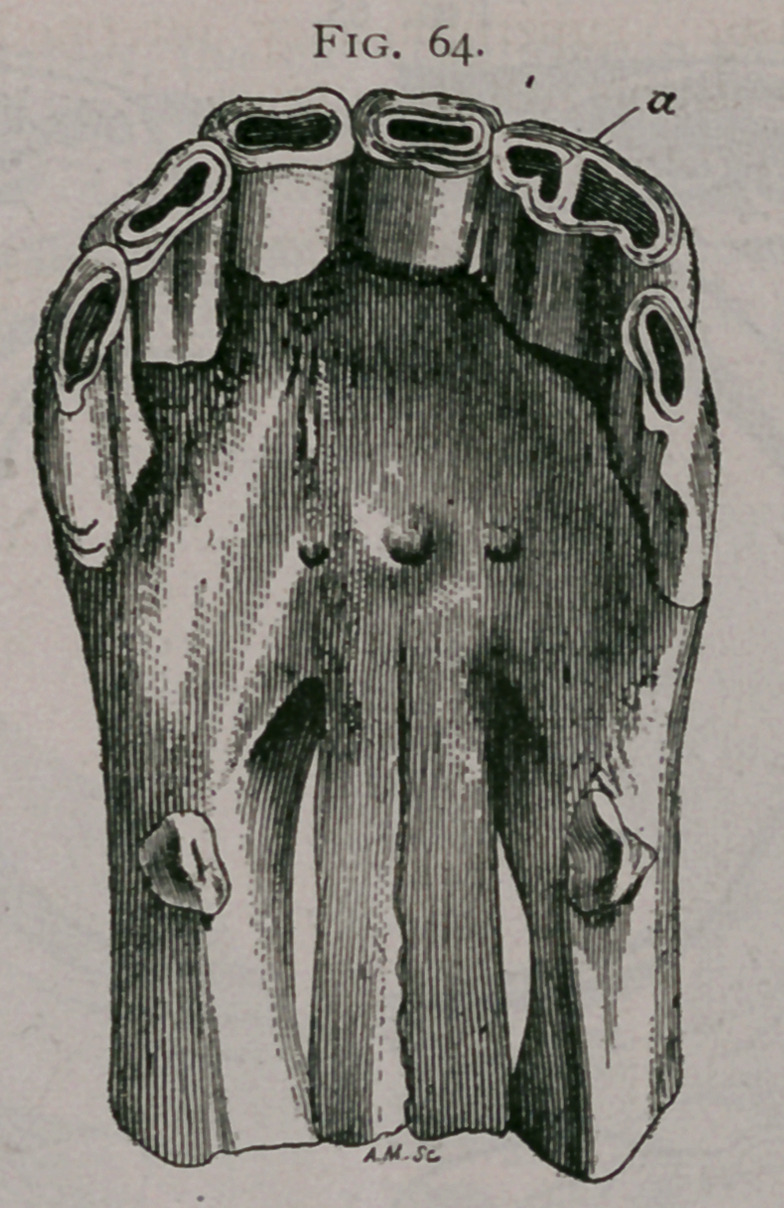


**Fig. 65. f5:**
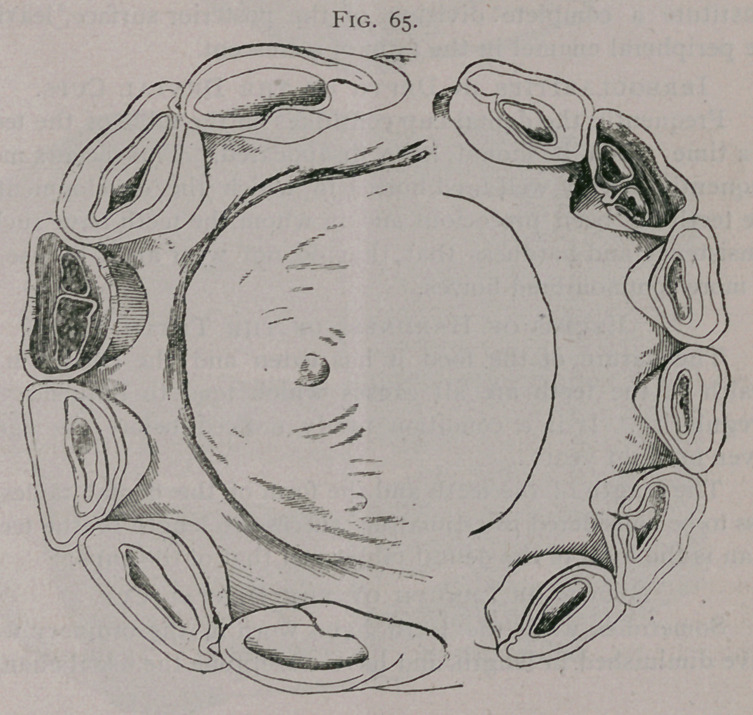


**Fig. 66. f6:**
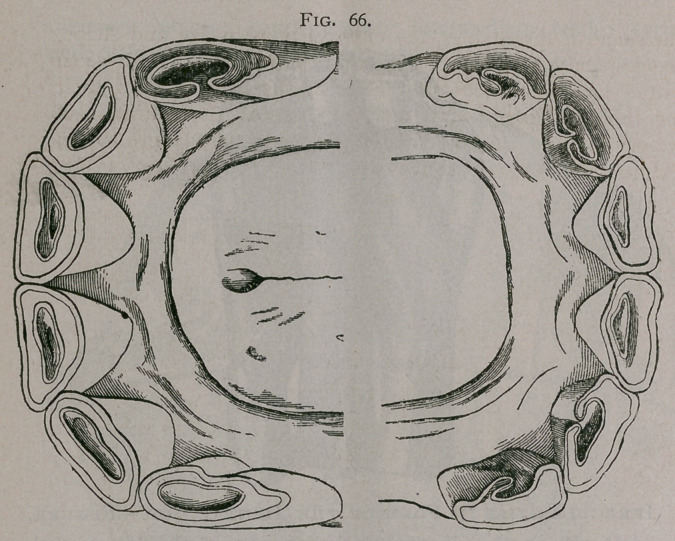


**Fig. 67. f7:**
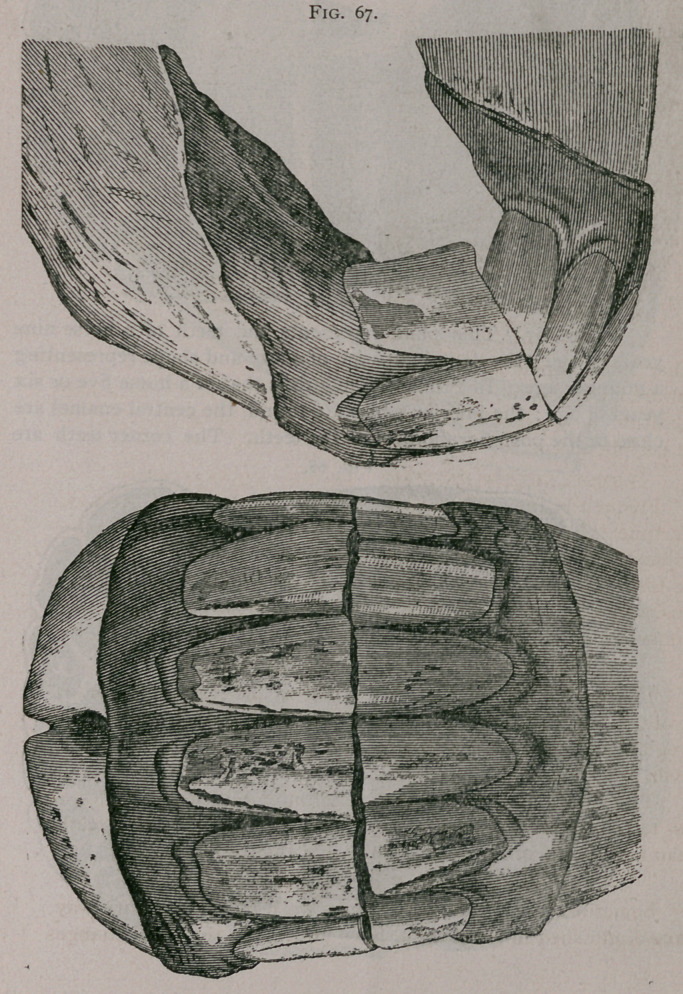


**Fig. 67. f8:**
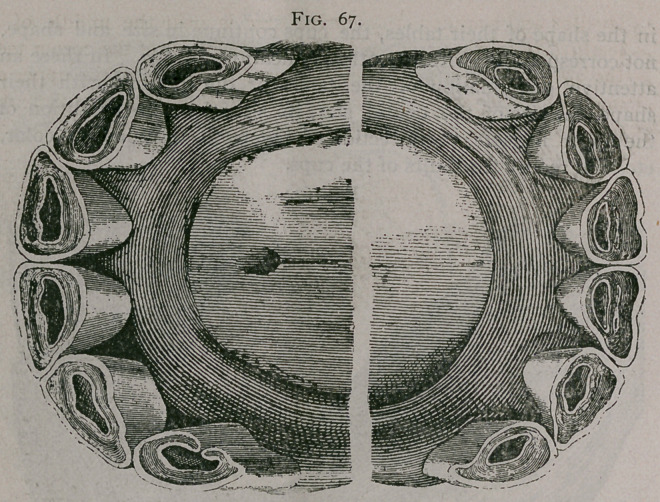


**Fig. 68. f9:**
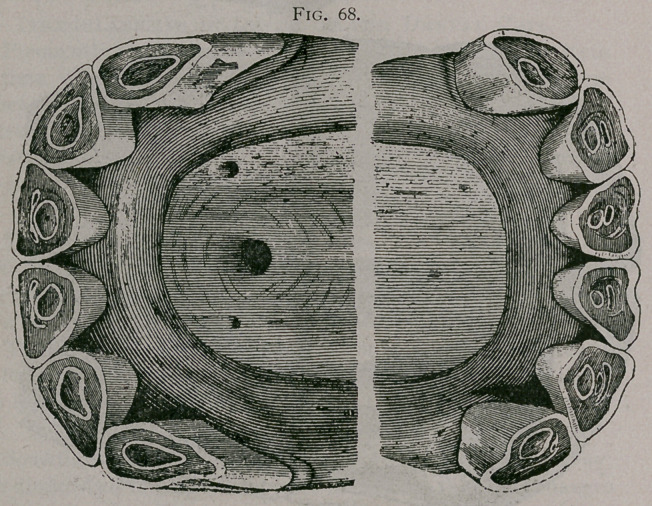


**Fig. 68. f10:**